# GPS Based Daily Activity Patterns in European Red Deer and North American Elk *(Cervus elaphus)*: Indication for a Weak Circadian Clock in Ungulates

**DOI:** 10.1371/journal.pone.0106997

**Published:** 2014-09-10

**Authors:** Erik P. Ensing, Simone Ciuti, Freek A. L. M. de Wijs, Dennis H. Lentferink, André ten Hoedt, Mark S. Boyce, Roelof A. Hut

**Affiliations:** 1 Chronobiology Unit, Centre for Behaviour and Neurosciences, University of Groningen, Groningen, the Netherlands; 2 Department of Biometry and Environmental System Analysis, University of Freiburg, Freiburg, Germany; 3 Department of Biological Sciences, University of Alberta, Edmonton, Canada; 4 Natuurmonumenten, Eenheid Veluwezoom, Rheden, the Netherlands; Simon Fraser University, Canada

## Abstract

Long-term tracking using global positioning systems (GPS) is widely used to study vertebrate movement ecology, including fine-scale habitat selection as well as large-scale migrations. These data have the potential to provide much more information about the behavior and ecology of wild vertebrates: here we explore the potential of using GPS datasets to assess timing of activity in a chronobiological context. We compared two different populations of deer (*Cervus elaphus*), one in the Netherlands (red deer), the other in Canada (elk). GPS tracking data were used to calculate the speed of the animals as a measure for activity to deduce unbiased daily activity rhythms over prolonged periods of time. Speed proved a valid measure for activity, this being validated by comparing GPS based activity data with head movements recorded by activity sensors, and the use of GPS locations was effective for generating long term chronobiological data. Deer showed crepuscular activity rhythms with activity peaks at sunrise (the Netherlands) or after sunrise (Canada) and at the end of civil twilight at dusk. The deer in Canada were mostly diurnal while the deer in the Netherlands were mostly nocturnal. On an annual scale, Canadian deer were more active during the summer months while deer in the Netherlands were more active during winter. We suggest that these differences were mainly driven by human disturbance (on a daily scale) and local weather (on an annual scale). In both populations, the crepuscular activity peaks in the morning and evening showed a stable timing relative to dawn and dusk twilight throughout the year, but marked periods of daily a-rhythmicity occurred in the individual records. We suggest that this might indicate that (changes in) light levels around twilight elicit a direct behavioral response while the contribution of an internal circadian timing mechanism might be weak or even absent.

## Introduction

Wildlife tracking using telemetry devices has become increasingly popular over the last decades [1]. More recently, satellite telemetry has allowed the collection of accurate locations even less than 1 minute apart [2]. Increased accuracy through global positioning system (GPS) allows for detailed, low biased sampling over long time intervals [3]. Many of these studies have used such data to evaluate spatial behavior and habitat selection of wild animals [2], [4], [5]. GPS location data have the potential to provide much more information about the behavior and ecology of wild vertebrates. Here, we used GPS tracking data to evaluate and compare chronobiological organization of year round daily activity patterns in two different populations of a large herbivorous mammal *(Cervus elaphus*, i.e., North American elk, and European red deer, hereafter generally referred as deer).

Most ungulates that live in temperate regions show crepuscular (twilight-active) daily activity pattern with two distinct peaks of activity in the twilights around dawn and dusk. This has been described, for instance, in red deer [6]–[8], roe deer (*Capreolus capreolus* [9], [10]), and mouflon (*Ovis aries* [11]). Ungulates kept in artificial or natural continuous darkness or continuous light, however, do not show clearly discernible daily activity rhythms [12]–[15]. Unfortunately, these kind of studies are limited by having to use either semi-domesticated animals, enclosed areas or small sample sizes. We aim to demonstrate an effective way to conduct such activity rhythm studies with the help of large sample sizes and long-term low-biased data collected with GPS tracking devices; these valuable data can be used to deduce daily activity patterns in free-ranging animals.

Activity rhythms in ungulates are of general interest to the field of chronobiology, because all species in this taxon are, almost without exception, fully exposed to changes in sunlight over the full 24-h per day. This poses the interesting hypothesis that ungulates may have lost the need for an internal representation of light intensity changes in the form a circadian clock. At least one empirical study support this hypothesis, given that arctic reindeer (*Rangifer tarandus plathyrhynchus*) seem to have lost the expression of a circadian clock [14], [16]. This observation has been attributed to the loss of day-night alterations of light intensity during the Arctic summer and winter. However, the finding that burrowing Arctic mammals like the Arctic ground squirrel (*Spermophilus parryii*) maintain strong diurnal variation in activity patterns and body temperature throughout the Arctic summer [17], [18] challenges the view that circadian rhythms are absent in the Arctic. This leads to an alternative hypothesis that burrowing animals have a strong need for an internal representation of the daily cycle in the form of a circadian clock, while non-burrowing animals like the ungulates may have lost the need for a circadian clock to regulate their activity patterns. This alternative hypothesis would predict that ungulates in temperate zones would also have weak circadian influence on their activity patterns, which should lead to relatively fixed phase angles between activity peaks and light intensity changes around twilight. An additional prediction would be that clear periods of a-rhythmicity may also be observed in ungulates living in temperate zones.

To test these predictions, we deduced daily activity patterns using GPS based transmitter data collected in two populations of the same species (*Cervus elaphus*) living in two completely different environments, i.e., red deer in the province of Gelderland, the Netherlands, and elk in the province of Alberta, Canada. To show that GPS based locomotor speed at a certain time can be used as an adequate measure for the activity at that time, we also compared the calculated activity profile of an animal to the activity profile based on head movements derived from activity sensors available for the same animal. Our secondary aim was thus to show how GPS-based locomotor speed is a good proxy for animal activity, thus opening new scenario for the study of chronobiology using GPS location data.

## Methods

### Ethics statement

Netherlands study: our data collection complied with all relevant national laws of the Netherlands. Procedures adopted in this study were reviewed and approved by the University of Wageningen Animal Experimentation Committee (Dierexperimenten Commissie, DEC; ALT 09-03). Natuurmonumenten is the proprietor of the national park Veluwezoom. Natuurmonumenten gave full consent for the execution of the field work in the Veluwezoom national park area.

Alberta study: our data collection complied with all relevant federal laws of Canada and provincial laws of Alberta. Procedures adopted in this study were reviewed and approved by the University of Alberta Animal Care and Use Committee ACUC – Biosciences (Animal care protocol # 536-1003 AR University of Alberta, Edmonton, Canada)and by all jurisdictions of the Alberta Government (Permit Numbers: BI-2008-19, RC-06SW-001 and 23181CN), and by Parks Canada (Permit Numbers: WL-2010-7292, WL-2010-5755).

### Netherlands study area

The study area in the Netherlands roughly covers 288 km^2^. The area is comprised of a number of smaller plots managed by private and government parties. Two of the larger plots are managed by Vereniging Natuurmonumenten, a Dutch institution for natural conservation, which provided GPS tracking data used for this study. These two areas are the Deelerwoud and National Park Veluwezoom, which consist of mixed deciduous and coniferous forest with large open spaces populated by heath and grasslands. The park is part of the larger Veluwe natural area and is part of the European ‘Natura 2000’ natural infrastructure plan. The park is located 5–10 km north-east of the city of Arnhem and receives a large number of visitors during the day. Parts of the park are enclosed by fences, mainly along railway lines and highways. The remaining plots have mixed uses, from forestry to agriculture. Red deer share the area with wild boar (*Sus scrofa*) and other wild ruminants, including Highland cattle (*Bos taurus*), fallow deer (*Dama dama*) and roe deer (*Capreolus capreolus*). There are no natural predators of red deer in the area and hunting is prohibited. However, the population size in the National Park is managed by Vereniging Natuurmonumenten through culling a yearly determined number of red deer. The total area is located between densely populated towns and villages and crossed by a major highway which is fenced on both sides. Overhead highway crossings for wild life (ecoducts) allow migration between National Park Veluwezoom and the rest of the Veluwe region.

### Canada study area

The study area in Alberta roughly covers 35.000 km^2^ and encompasses a diverse range of biotopes and land uses. It is situated on the eastern slopes of the Rocky Mountains and varies from flat agricultural grasslands to mixed deciduous and coniferous forests. Some of the monitored elk moved to southeastern British Columbia and northwestern Montana during the study.

The area encompasses three different land uses; 1) Public lands, where all types of recreational activities are allowed (e. g. camping, All Terrain Vehicles) and hunting is permitted to any licensed hunter; 2) Private lands, where activities are similar to those recorded on public lands but restricted by landowners; and 3) National Parks (Waterton Lakes in Alberta and Glacier in Montana, USA) where hunting is prohibited. The area hosts a wide variety of other large herbivores including moose

(*Alces alces*), white-tail Deer (*Odocoileus virginianus*) and mule Deer (*Odocoileus hemionus*). There is also a wide range of native large predators including cougar (*Puma concolor*), wolf (*Canis lupus*), coyote (*Canis latrans*), black Bear (*Ursus americanus*) and grizzly Bear (*Ursus arctos*).

### Data sets

The GPS datasets from National Park Veluwezoom consisted of 11 red deer (6 female, 5 male), which were monitored for various duration between 2010 and 2013.

The GPS data from Alberta were from 180 elk (118 female, 62 male), divided into 7 herds according to capture location. Scatter plots of the raw coordinates of individual deer were used to validate the designated herds grouping. Capture location was a clear predictor for herd designation in females, but not in males. This confirms the known social organization of red deer and elk, where mainly the females form a stable herd structure, while males (especially younger ones) often disperse [19], [20]. This analysis also revealed that the Crowsnest and Livingstone herds had a high instance of overlapping, which is why we subsequently combined the two herds to one named Crowsnest/Livingstone.

Of the 181 total deer we selected 46 (31 female, 15 male) with a minimum dataset length of one year, trying to use deer from each herd equally. The number of deer that were followed for a year or more was not equal in each herd and one herd (Porcupine Hills) had to be discarded because it only contained 3 useable animals. The herd Whaleback had only one male that was followed for more than a year and thus was not analyzed. See *table 1* for full details on sample size deployed in our analyses.

**Table 1:** Number of deer tracked for each herd.

	Veluwezoom	Carbondale	Porcupine	Beauvais	Livingstone	Crowsnest	Waterton	Whaleback
Total	12	72	8	9	22	17	23	30
>1 y. recorded	10	56	3	6	7	10	8	11
Analyzed	11	13	0	6	11		7	10
Female	6	7	0	4	6		4	10
Male	5	6	0	2	5		3	0

Deer sample sizes monitored from different herds in Veluwezoom and Alberta. Variables presented are: *total* - number of deer equipped with GPS logger for each herd; *> 1y. recorded* – number of deer that were recorded for more than one year; *analyzed* – total number of deer from which the GPS data were analyzed; *female/male* – number of female/male deer from which GPS data were analyzed. Porcupine Hills only had three deer that were tracked for more than one year and were therefore not analyzed. Livingstone and Crowsnest overlapped heavily in their home ranges and were treated as a single herd. Waterton and Whaleback both had one animal which had to be discarded due to erratic GPS locations. We only analyzed thirteen representative animals of the Carbondale herd to avoid over representation of this herd relative to the other herds analyzed.

### Capture procedure the Netherlands

To mount the GPS transmitters collars (Vectronic Aerospace GmbH), the deer in the Netherlands were anaesthetized with Etorphine (2,25 mg/ml) using a tranquillizer gun (Daninject, 1cc Etorphine/50 kg) at a distance of between 30 and 40 meters while the animals were congregated around a feeding trough. After mounting the transmitter collar the deer were revived by administering a counter agent (Naltrexone, 50 mg/ml, administered intravenous at ½ times the volume of Etorphine administered). The entire procedure took 30–90 min. The collars were programmed with a 1-h sampling interval. Data was transmitted through cell phone communication service.

### Capture procedure Canada

Elk were captured during the winters of 2007–2012 using helicopter net-gunning. Females were fitted with Lotek GPS-4400 or GPS-4500 radio telemetry collars (Lotek wireless Inc., Ontario, Canada), whereas males were fitted with Lotek ARGOS GPS radio telemetry collars. All collars were programmed with a 2-h relocation schedule. Data of females were remotely downloaded in the field, whereas satellite transmitted data of males were received monthly via email.

### Calculations

To assess speed of locomotion (*S_AB_*), we first calculated the distance between two coordinates, accounting for the curvature of the earth, using eq*uation 1* [21]

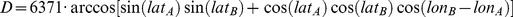
(eq.1)where *D* is the horizontal distance travelled between two mapped coordinates (*A* and *B*) in kilometers, *lat* is the latitude in radials and *lon* is the longitude in radials. To compensate for inaccuracies in the measurement of the GPS coordinates by the satellite we set all travelled distances less than 3 m to 0 m travel distance.

We subsequently calculated the true travel distance corrected for average change in altitude between the two coordinates using *equation 2*, 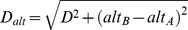
(eq.2)where *D_alt_* is the true distance travelled in kilometers, accounting for changes in altitude, *D* is the horizontal travel distance as calculated in equation 1 and *alt* is the altitude (in m) in coordinate *A* or *B*. Finally, we calculated the speed of the animals between the two measurements, *A* and *B* using *equation 3*

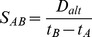
(eq.3)where *S_AB_* is the average speed between two coordinates *A* and *B* in km/h, *D_alt_* is the altitude corrected travel distance in km as calculated in equation 2 and *t* is the time stamp of the GPS coordinate determination in *A* and *B* in hours.

To calculate civil and nautical twilight times throughout the years of study we used a day length calculator algorithm provided by the US *National Oceanic and Atmospheric Administration* (NOAA) [22]. Both nautical and civil twilight were used to categorize timing of activity. Nautical twilight is defined as the time when the sun is between 12 and 6 degrees below the horizon, civil twilight is defined as the time when the sun between 6 and 0 degrees below the horizon. During these twilights, 80% of the change of sunlight intensity takes place, while the change in sun light intensity is maximal when the sun is 6 degrees below the horizon (at the start of civil twilight at dawn and the end of civil twilight at dusk) [23], [24].

### Activity percentages

To describe daily activity patterns, the occurrence of activity was labeled as day time activity, night time activity, or twilight activity. We operationally defined ‘Twilight’ as the time between the start of nautical twilight in the morning until sunrise and sunset until the end of nautical twilight in the evening. ‘Day’ was defined as the time between sunrise and sunset and ‘Night’ was defined as the time between the end of nautical twilight in the evening and the start of nautical twilight in the morning.

### Activity indices and phase angle differences (Ψ)

To quantify the relative amount of activity during the light phase of the day we calculated a diurnality index in which we defined the light phase of the day as time between the start of civil twilight in the morning and the end of civil twilight in the evening. The ‘night’ was defined as the time between the end of civil twilight in the evening and the start of civil twilight in the morning. For the calculation of the crepuscularity index we defined ‘twilight’ as the time between the start of nautical twilight in the morning until sunrise and sunset until the end of nautical twilight in the evening. We used *equation 4* and *5* to calculate the indices:
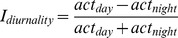
(eq.4)

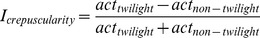
(eq.5)where *I_diurnality_* is the diurnality index, *I_crepuscularity_* is the crepuscularity index, *act_day_* is the total activity/h in the light phase, *act_night_* is the total activity/h in the dark phase of the day, *act_twilight_* is the total activity/h during the twilights (intervals nautical twilight-sunrise and sunset-nautical twilight) and *act_non-twilight_* is the total activity/h during the remainder of the day.

Phase angle differences (Ψ) between activity peaks and the onset and offset of civil twilight were calculated as end of civil twilight at dawn minus timing of morning activity peak and beginning of civil twilight at dusk minus timing of dusk activity peak. Negative Ψ indicates that the activity peak lags behind the onset or offset of civil twilight.

### Locomotor speed as a proxy for activity: comparison of methods

To compare locomotor speed with another indicator of activity, we calculated Pearson correlation coefficients between GPS based locomotor speed (*D_alt_*) and activity data obtained from activity sensors placed in the GPS collars of ten Canadian deer from the Crowsnest/Livingstone herd.

## Results

**Figure 1:**
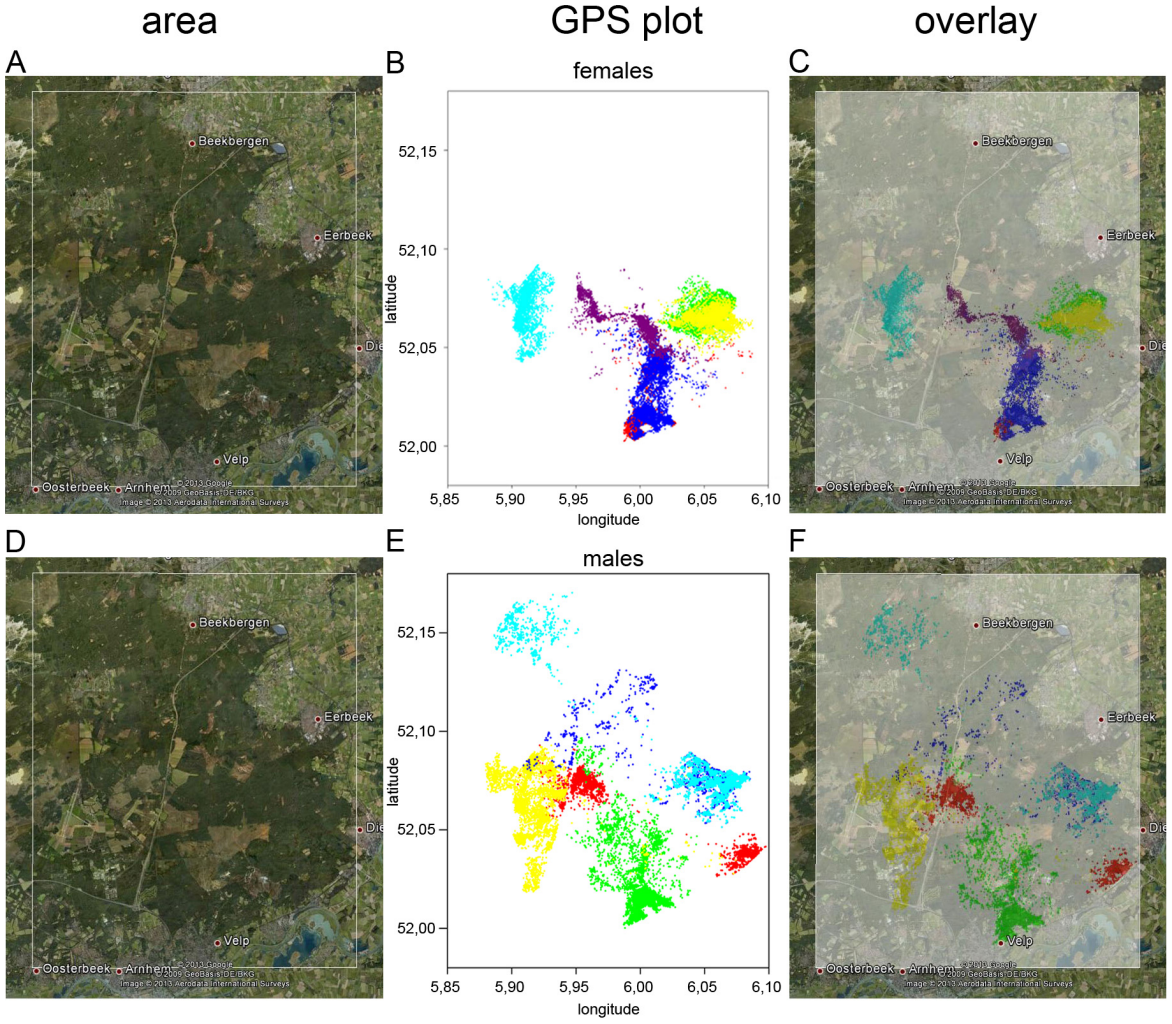
Spatial distribution map of red deer in the Veluwezoom. Overlay of Google Earth derived satellite images with scatter plots of GPS locations for each individual. Different colors represent different individuals.

**Figure 2:**
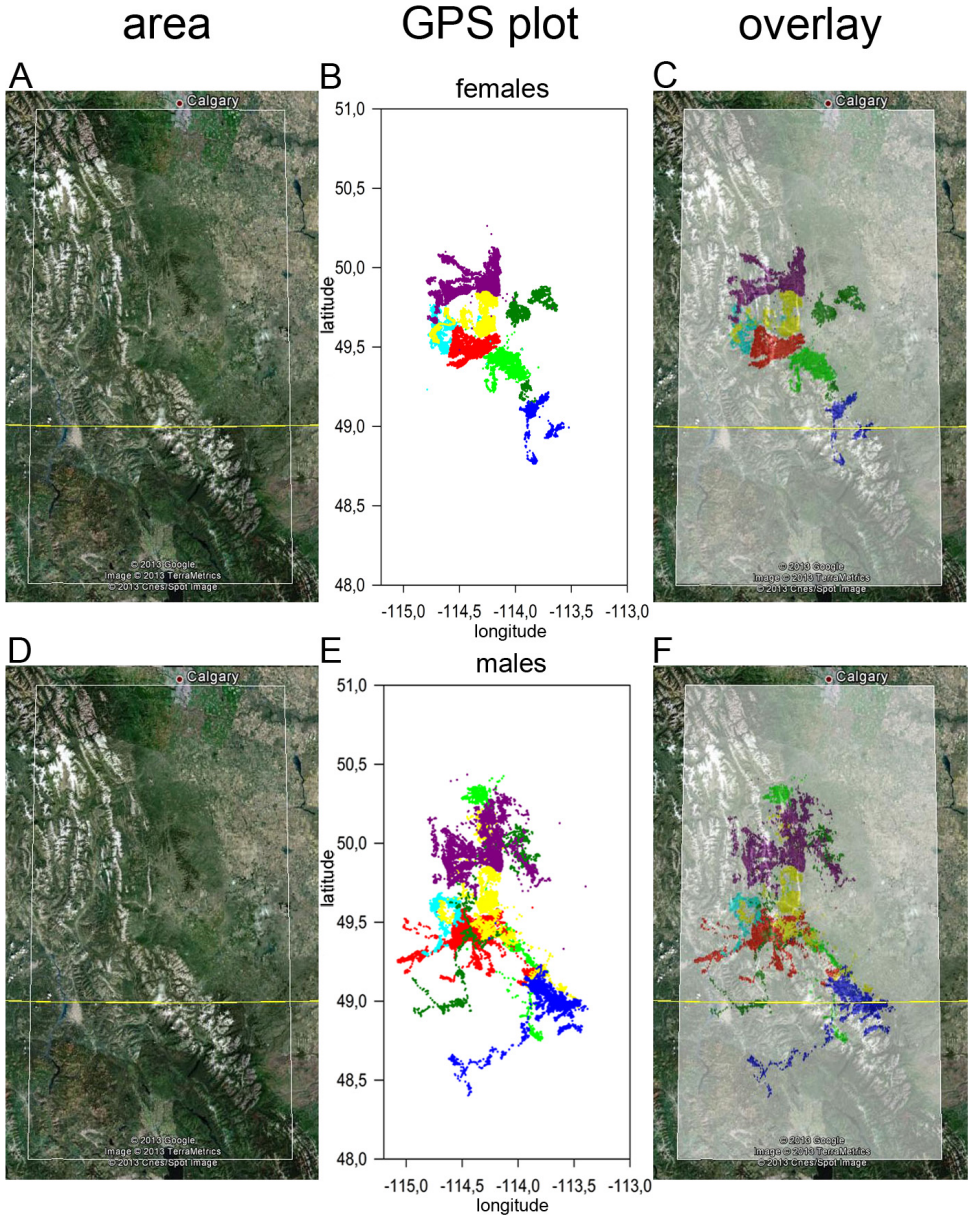
Spatial distribution map of red deer in Alberta. Overlay of Google Earth derived satellite images with scatter plots of GPS locations for individuals within each herd. Different colors represent different herds: Waterton (dark blue), Porcupine Hills (dark green), Carbondale (red), Beauvais (light green), Crowsnest/Livingstone (yellow and light blue) and Whaleback (purple).

An overview of the original GPS locations per individual used in this study shows that deer in the Veluwezoom area in the Netherlands (Fig.1) use roughly 1/10^th^ the size of home ranges used by Canadian deer (Fig.2). This suggests that deer in the Veluwezoom area are probably more limited in their movements by human terrain use and disturbances.

### Actograms

**Figure 3:**
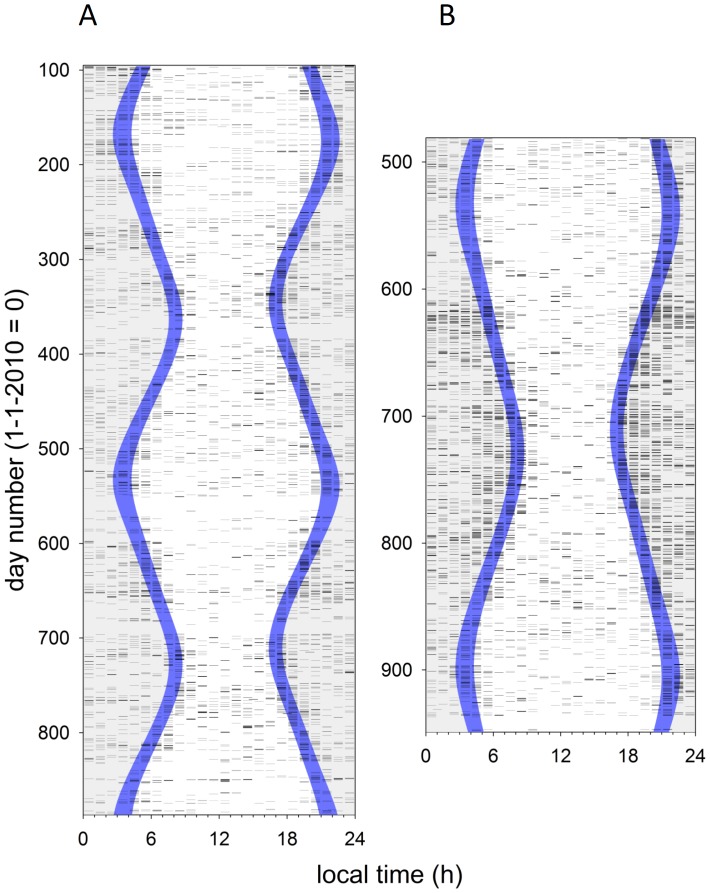
Actograms of a female (A) and a male (B) red deer from the Veluwezoom. The vertical axis represents the number of days since 1-1-2010, the horizontal axis represents the time of day. The thickness of each black bar represents the average speed of locomotion per hourly interval. The blue area represents the time between nautical twilight start at dawn and sunrise, or between sunset and nautical twilight end at dusk. For other actograms of deer from the Veluwezoom, see Fig.S1–Fig.S9.

At the individual level, crepuscular activity patterns are quite variable and are interchanged with marked periods without apparent daily rhythmicity in locomotor activity for both male and female red deer (Fig.3; for other actograms see Fig.S1–Fig.S9). Also the total amount of daily activity was found to be quite variable throughout the year. The sampling frequency of one or two samples per hour did not allow for studying faster rhythms in the ultradian range.

### Activity profiles

**Figure 4:**
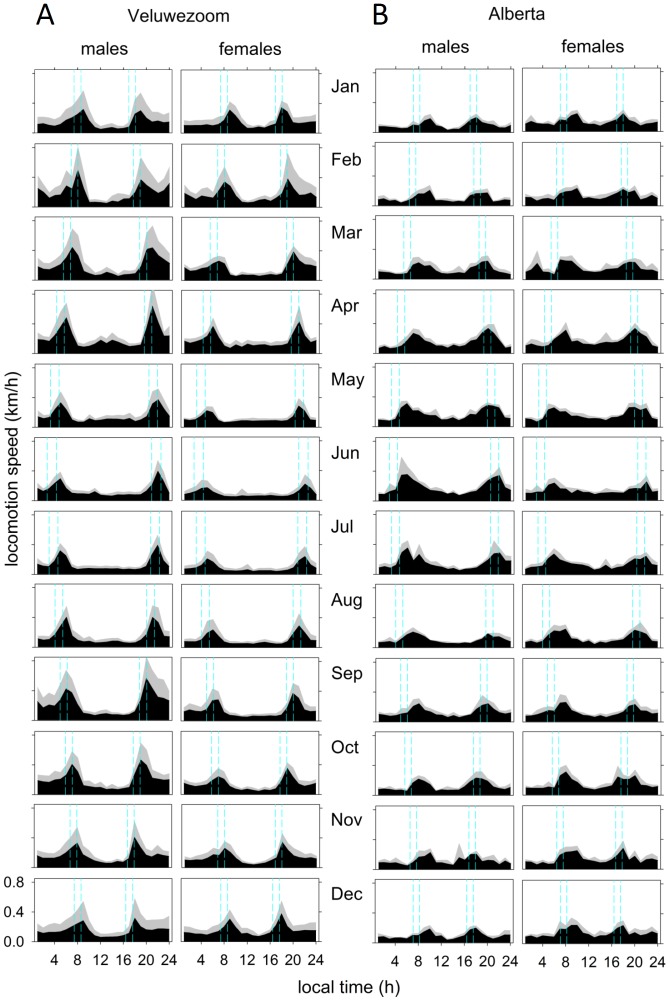
Activity profiles of male and female red deer in the Veluwezoom (A) and Alberta (B). Black areas represent activity, grey areas represent standard deviation. Blue dotted lines indicate start of nautical twilight, sunrise, sunset and end of nautical twilight.

To assess the average pattern of activity for male and female deer in each study population, we calculated average daily activity profiles per month in males and females for each study area (Fig.4). These profiles show two peaks in activity, during and shortly after twilight periods at dawn and at dusk. Although both populations show this crepuscular activity profile, the dawn and dusk peaks were more pronounced in the Veluwezoom deer. The clearest crepuscular activity pattern was observed in Veluwezoom males in spring. The weakest crepuscular activity profile is seen in Alberta deer in winter.

### Phase angles

**Figure 5:**
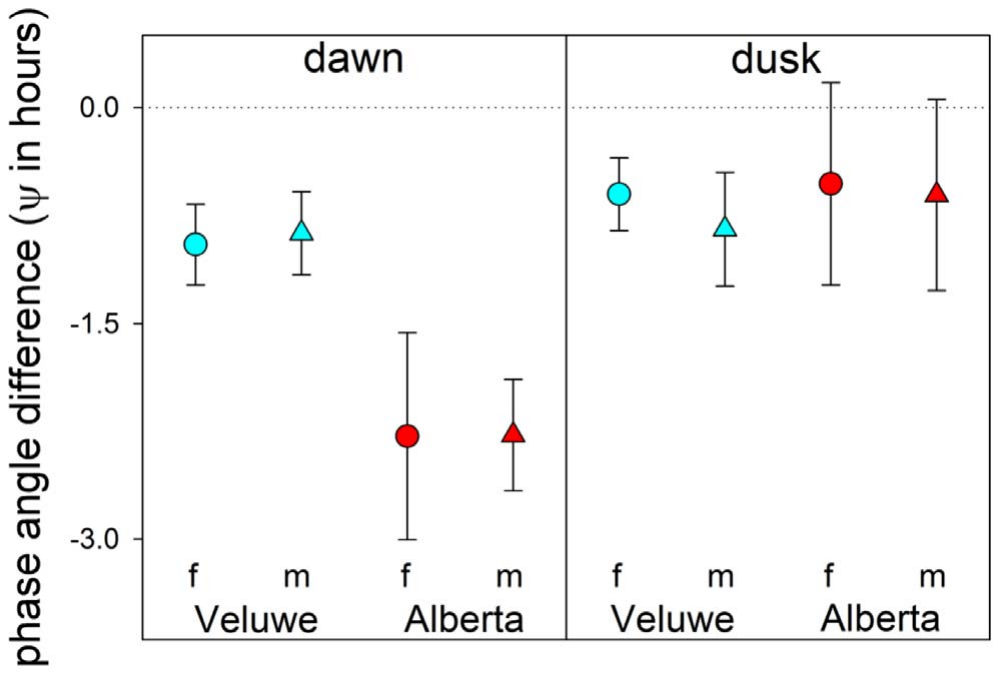
Average phase angle difference over a year. The negative phase angle differences (Ψ in hours) indicate that the activity peak consistently lags behind civil twilight onset at dawn and the civil twilight offset at dusk. Dawn activity peaks later in Canadian deer than in deer from the Netherlands. Circles represent the females and the triangles represent the males. The deer from the Netherlands are in blue and the Canadian deer are in red. The error bars show standard deviation from the mean. Dawn onset and dusk offset of civil twilight is set as zero (dotted line).

The activity profiles indicate that the phase angle of the activity peak relative to dawn and dusk (Ψ) was stable throughout the year as the peak shifted completely with the seasonal change of twilight times. The crepuscular activity peaks always lagged behind the timing of civil twilight onset in the morning and civil twilight offset in the evening (negative phase angle, Fig.5). This lag was most pronounced in both female and male Canadian deer, where the activity peak was almost two hours after civil twilight in the morning (Fig.5). These data strongly suggest that the daily activity peaks may reflect a response to the fast change in light intensity at dawn civil twilight onset and dusk civil twilight offset, rather than anticipation of twilight driven by an internal circadian timing mechanism.

### Activity distribution over the day

**Figure 6:**
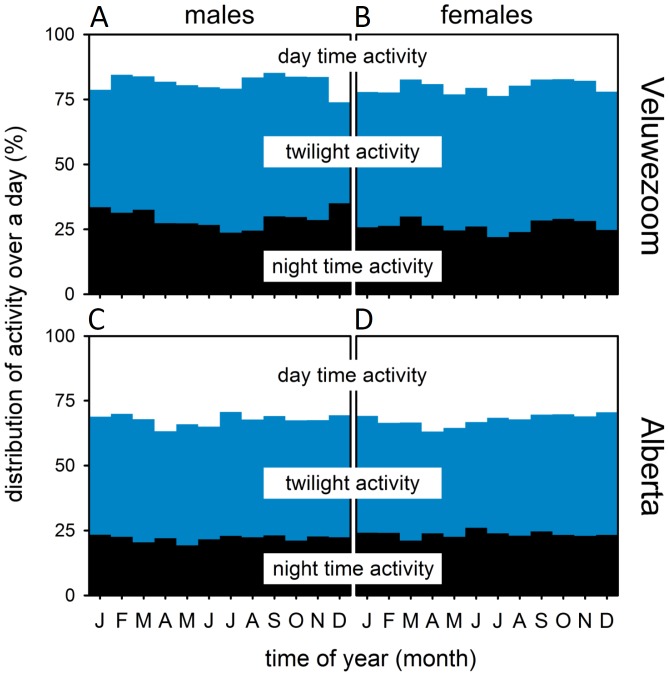
The distribution of activity over the day for each month. Activity distributions were calculated for Veluwezoom (A, B) and Alberta (C, D), for males (A, C) and females (B, D). Percentage of activity was calculated as part of the total distance travelled during the 24 hours. The white portions of the bar represent fraction of activity during the day (between sunrise and sunset), the blue portions the fraction during twilight (between nautical twilights and sunrise/sunset) and the black portion the fraction during the night (between nautical twilight end at dusk and nautical twilight start at dawn).

Because of the marked peaks in the activity profiles we plotted the percentage of average distance travelled per hour within each of 3 separate parts of the 24 h day: day, night and twilight. These plots show that the deer showed similar activity levels at all phases of the 24 h day, albeit that travel distances are greater during the twilight periods (Fig. 6).

**Figure 7:**
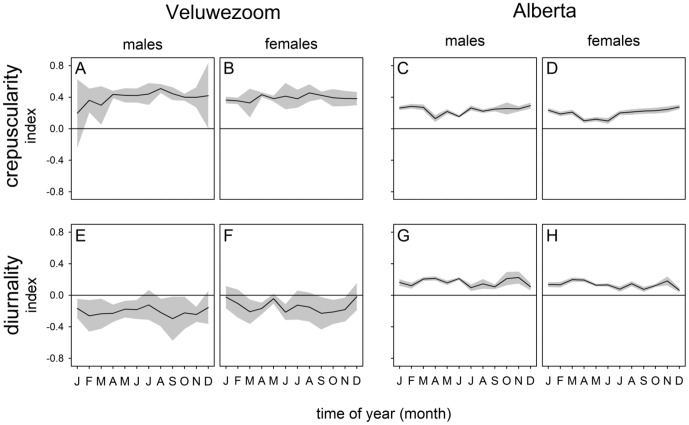
Preference for twilight activity or diurnal activity. Crepuscularity (A–D) and diurnality (E–F) indices for male (A,C,E,G) and female (B,D,F,H) red deer in the Veluwezoom (A,B,E,F) and Alberta (C,D,G,H). The black line represents the average index value, the grey areas represent the 95% confidence interval, which can be interpreted as a one-sided T-test: when the 95% confidence interval (grey area) does not encompass the 0 line, the activity pattern significantly deviates from an equal distribution (p<0.05).

To assess whether the deer in the two populations differ in their preference for nocturnal, diurnal or twilight activity, we calculated diurnality and crepuscularity indices which can vary between -1 and 1. A positive value for the diurnality index indicates a diurnal preference whereas a positive value of for the crepuscularity index indicates a preference for activity around civil twilight. Overall males and females did not differ in their distribution of activity. Throughout the year, both populations have a preference for twilight activity (Fig.7 A–D). A marked difference between both populations can be found in the diurnality index, where the Veluwezoom population shows a clear preference for nocturnal activity, the Alberta population prefers to be more active during the day (Fig.7 E–H).

### Comparison of methods

To evaluate whether speed is an accurate measure for activity we analyzed data from ten Canadian deer of which both GPS position and head movements were recorded simultaneously. Correlations between both (log transformed) measures of movement were highly significant (p<0.0001) in all deer and correlation coefficients (*r*) ranged between 0.36 and 0.73 (table 2). This indicates that both activity proxies are probably suitable as measures for general activity in red deer, albeit that the recording of head movements is likely to be more sensitive during grazing when locomotor speed is likely to be low. We conclude that GPS data form a reliable source of data for reconstructing daily locomotor activity profiles in ungulates.

**Table 2:** Correlations between head movements and locomotor speed.

id	Constant	coefficient	r	p	n_20%_
27	−1.96	0.018	0.36	<0.0001	840
82	−2.32	0.034	0.67	<0.0001	828
95	−1.94	0.048	0.47	<0.0001	747
113	−2.06	0.031	0.57	<0.0001	741
132	−2.12	0.029	0.64	<0.0001	624
137	−2.26	0.042	0.66	<0.0001	748
139	−2.14	0.035	0.63	<0.0001	751
146	−2.75	0.148	0.70	<0.0001	832
158	−2.31	0.043	0.73	<0.0001	686
160	−2.26	0.042	0.70	<0.0001	865

Pearson regression coefficient between total GPS logger movement (measured by head movements) and average locomotor speed (D_alt_ in km/h, eq.3) for 2 h epochs were performed to assess correlation coefficients in 10 Canadian deer. To avoid autocorrelation effects due to sequential interdependency in these time series, we randomly selected 20% of the data for the calculations (on average resulting in 1 data pair every 10 h). Square root transformations on both variables were performed to reach normal distributions.

## Discussion

The comparison of head movements versus speed of locomotion showed that both methods can be used as a proxy for daily activity patterns in large herbivores. GPS based activity data do not rely on small bodily movements (*e.g.* grazing, grooming) as is the case with head movement data. GPS data are therefore more indicative of activity patterns on longer time scales (hours) and therefore more relevant for circadian and metabolic evaluation. The overall bimodal activity pattern of GPS based data is in agreement with the activity patterns previously found in ungulates using activity sensors [13], [14], [25]. GPS tracking therefore provides reliable data with little bias for chronobiological activity patterns in other wild animal species, requiring a minimal sampling frequency of one bearing every one or two hours. More frequent sampling increases reliability and accuracy of the daily activity patterns obtained and may open the possibility to study ultradian activity patterns which may be important for feeding cycles in ruminants.

In general, red deer in both populations were found to have crepuscular activity rhythms with the highest level of locomotor activity taking place in the first hours after civil twilight onset at dawn and civil twilight offset at dusk. Although males in both populations covered larger home ranges than females, their average daily activity patterns were similar. A marked difference between both populations was found in the fact that the Canadian deer showed a preference for diurnal activity, while the deer in the Netherlands showed a nocturnal preference. Another difference between the two populations is the time of year when they were most active. Deer in the Netherlands were found to be most active in winter while Canadian deer were most active in summer. The aforementioned difference in activity between Canadian deer and deer in The Netherlands is likely to be caused by avoidance of human disturbance, which is higher during the day and during the summer. Although the area is inhabited by locals, visitors in the park area are only allowed at daylight hours, which may have led to the higher degree of nocturnality in deer in the Netherlands, especially in summer. Several studies have shown that red deer change their behavior to avoid human disturbance by, for example, grazing further away from busy roads, so it seems probable that human disturbance may also change their daily activity patterns if the disturbance is prolonged and constant [9], [26]–[29]. Another factor that may have contributed to the higher degree of nocturnality in the Netherlands and diurnality in Canada is the presence of natural predators in Canada (and their absence in The Netherlands), which can be expected to be mainly nocturnal in their activity patterns [30].

The difference between the two populations in terms of their most active season may also arise from climatic conditions. The Dutch population seems to be most active in the winter months as the winters in The Netherlands are mild and do not have prolonged periods of extreme cold or heavy snowfall and their sources of food are accessible throughout the year. In combination with more human disturbance during summer, this likely led to more intense foraging in the winter months without strong preference for nocturnal or diurnal activity (Fig.7). In Canada, conversely, the winters are harsh with very low temperatures and high snowfall, making successful foraging a challenge. During the warm summers human disturbance is also at its peak in Canada, but compared to the density of the human population in the Netherlands that is likely to be of much lower impact.

From the activity profiles (Fig.4) and the phase angles of the dawn and dusk activity peaks (Fig.5), we can conclude that red deer and elk likely have a weak internal clock as their activity peaks precisely followed (and did not anticipate) the twilight times over the year. Species with a stronger circadian clock typically show a limit in their ability to adapt to changes in photoperiod [23]. In arctic reindeer [13], [14] as well as in the horse [31], behavioral and physiological data suggest that ungulates may generally have weak circadian organization. It therefore seems likely that the daily activity peaks around dawn and dusk are directly driven by the 1–1.5 hours of twilight at dawn and dusk while the contribution of a circadian timing mechanism seems limited. This conclusion suggests that the lack of circadian organization in behavioral rhythmicity in ungulates may not be limited to arctic regions.

## Supporting Information

Figure S1 **Actogram of Veluwezoom male 8765.** See Fig.3 for explanation.
(TIF)

Figure S2 **Actogram of Veluwezoom male 8833.** See Fig.3 for explanation.
(TIF)

Figure S3 **Actogram of Veluwezoom male 8835.** See Fig.3 for explanation.
(TIF)

Figure S4 **Actogram of Veluwezoom male 9470.** See Fig.3 for explanation.
(TIF)

Figure S5 **Actogram of Veluwezoom female 6053.** See Fig.3 for explanation.
(TIF)

Figure S6 **Actogram of Veluwezoom female 8762.** See Fig.3 for explanation.
(TIF)

Figure S7 **Actogram of Veluwezoom female 9385.** See Fig.3 for explanation.
(TIF)

Figure S8 **Actogram of Veluwezoom female 9467.** See Fig.3 for explanation.
(TIF)

Figure S9 **Actogram of Veluwezoom female 9471.** See Fig.3 for explanation.
(TIF)
