# Supplementary figures and images for: GPS Based Daily Activity Patterns in European Red Deer and North American Elk (Cervus elaphus): Indication for a Weak Circadian Clock in Ungulates
Source: PLoS One. 2014 Sep 10;9(9):e106997. doi: 10.1371/journal.pone.0106997 (PMC4160215; doi:10.1371/journal.pone.0106997)

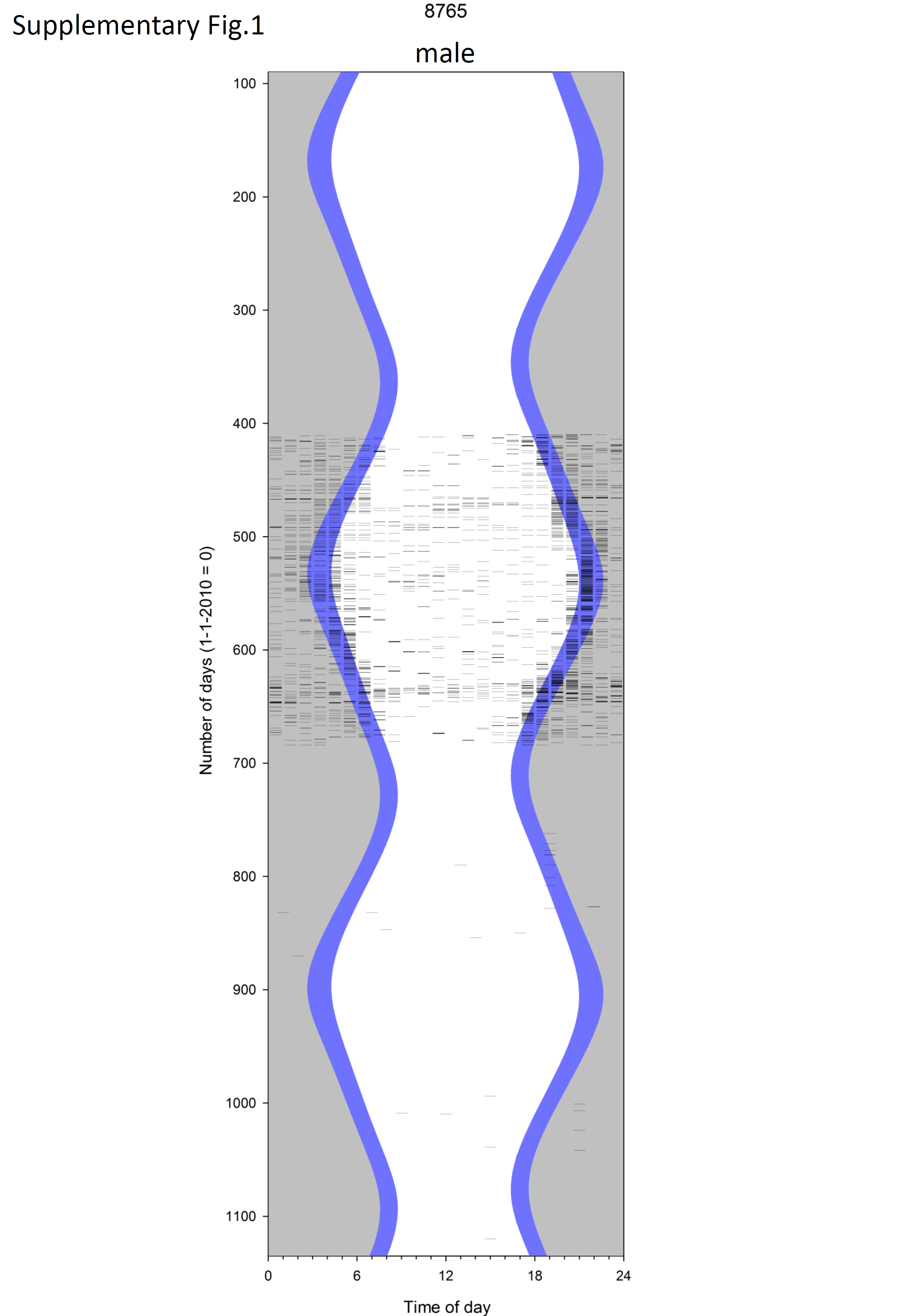

Supplement: Figure S1 — Actogram of Veluwezoom male 8765. See Fig.3 for explanation. (TIF) [file pone.0106997.s001.tif]

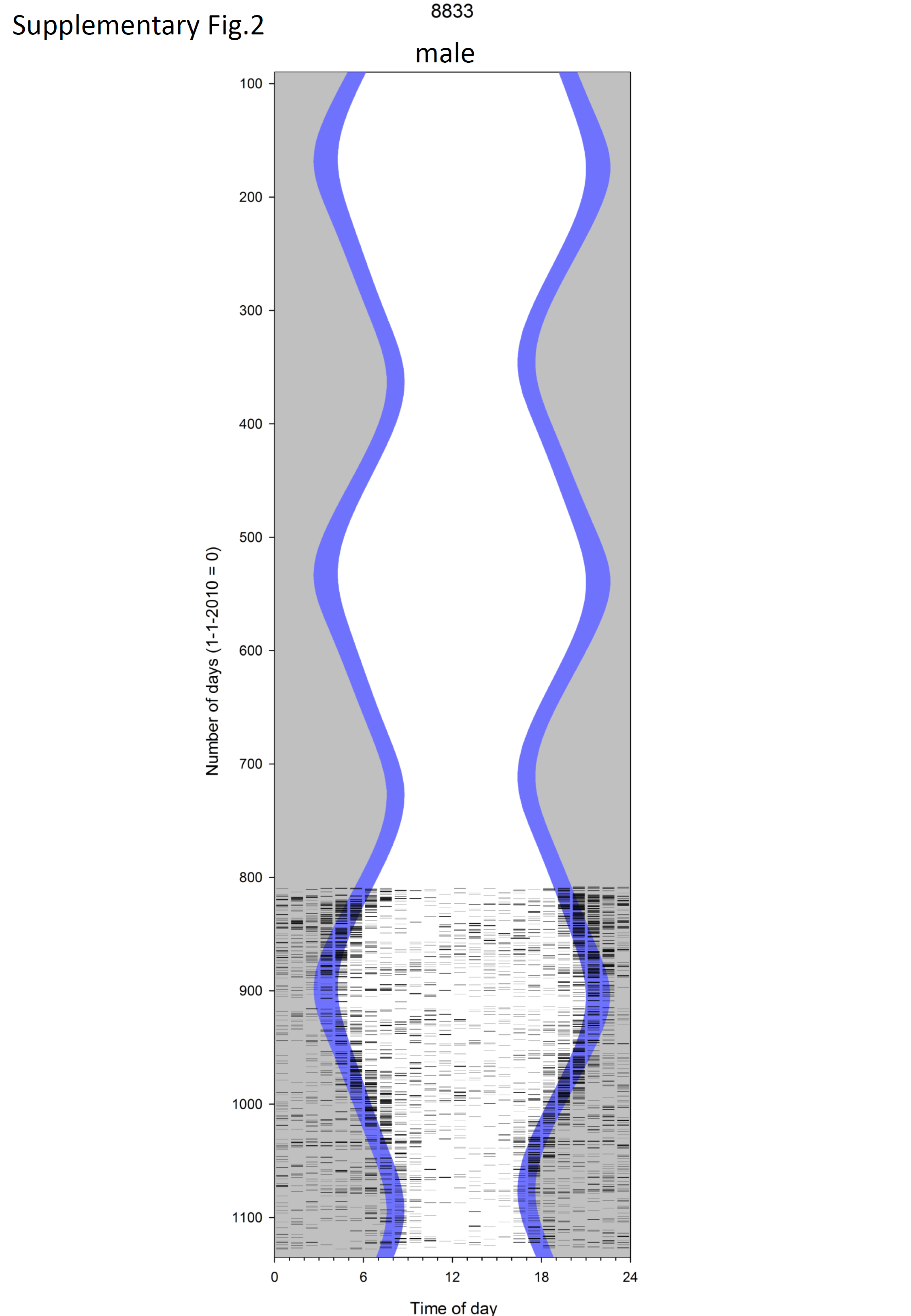

Supplement: Figure S2 — Actogram of Veluwezoom male 8833. See Fig.3 for explanation. (TIF) [file pone.0106997.s002.tif]

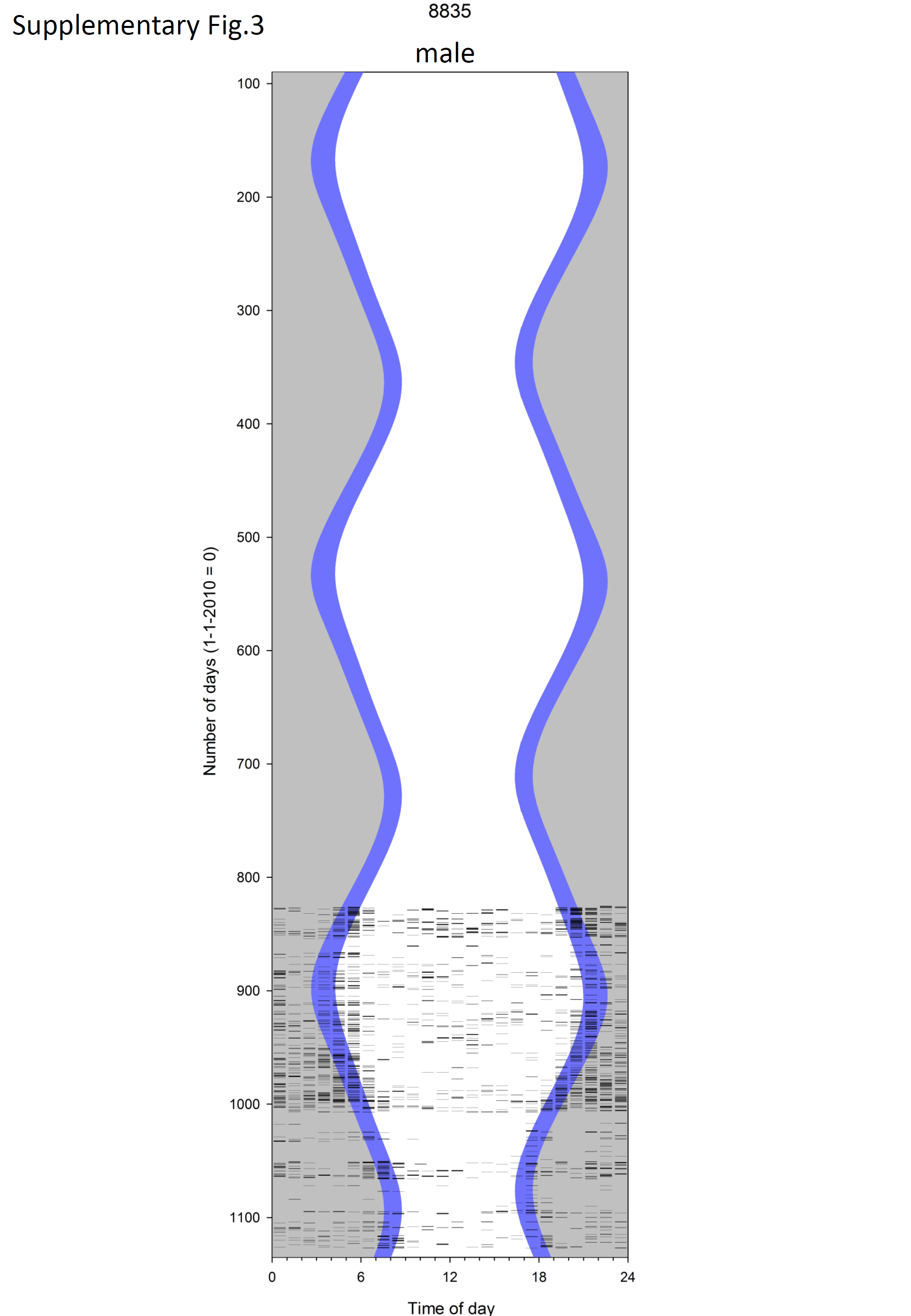

Supplement: Figure S3 — Actogram of Veluwezoom male 8835. See Fig.3 for explanation. (TIF) [file pone.0106997.s003.tif]

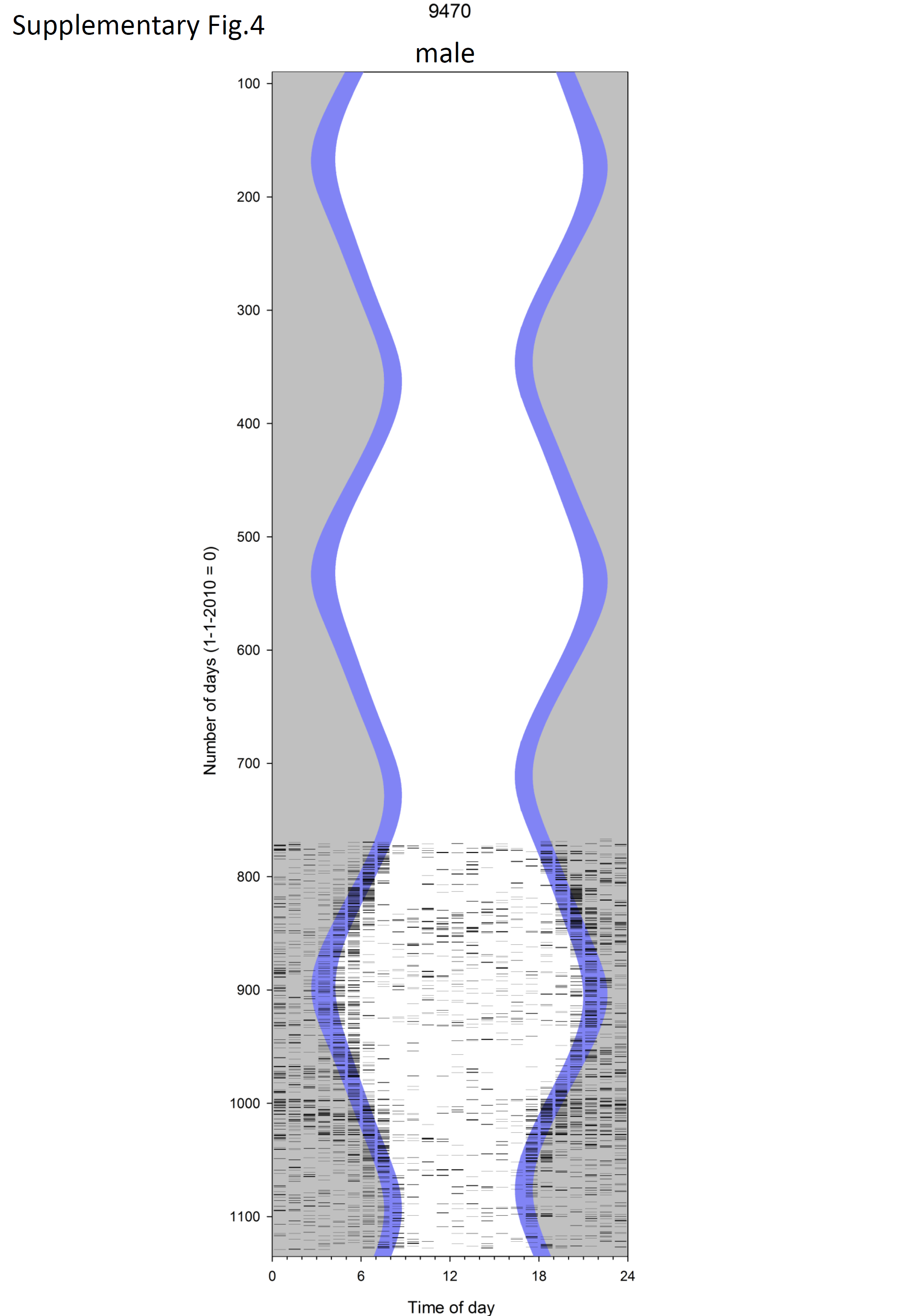

Supplement: Figure S4 — Actogram of Veluwezoom male 9470. See Fig.3 for explanation. (TIF) [file pone.0106997.s004.tif]

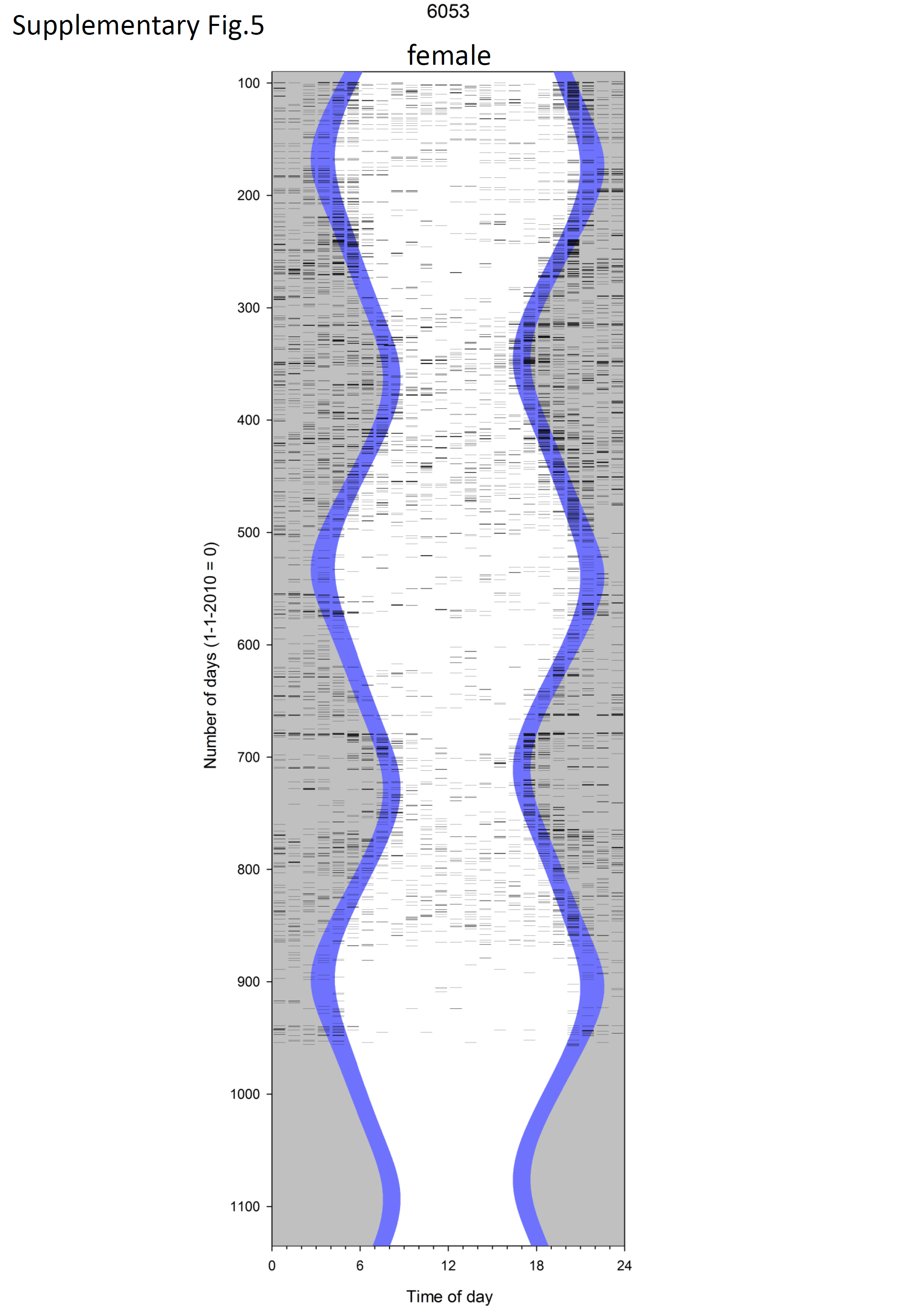

Supplement: Figure S5 — Actogram of Veluwezoom female 6053. See Fig.3 for explanation. (TIF) [file pone.0106997.s005.tif]

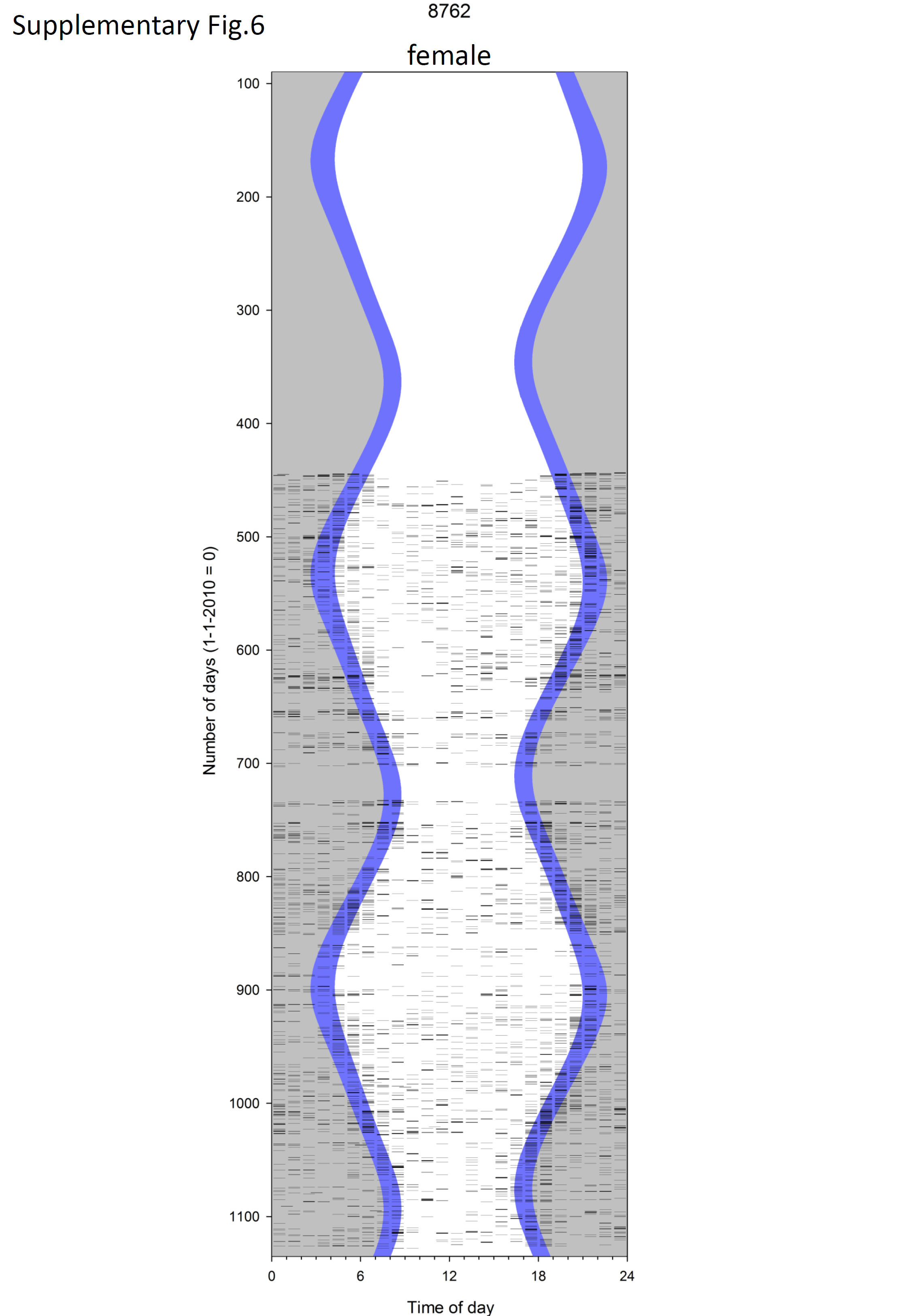

Supplement: Figure S6 — Actogram of Veluwezoom female 8762. See Fig.3 for explanation. (TIF) [file pone.0106997.s006.tif]

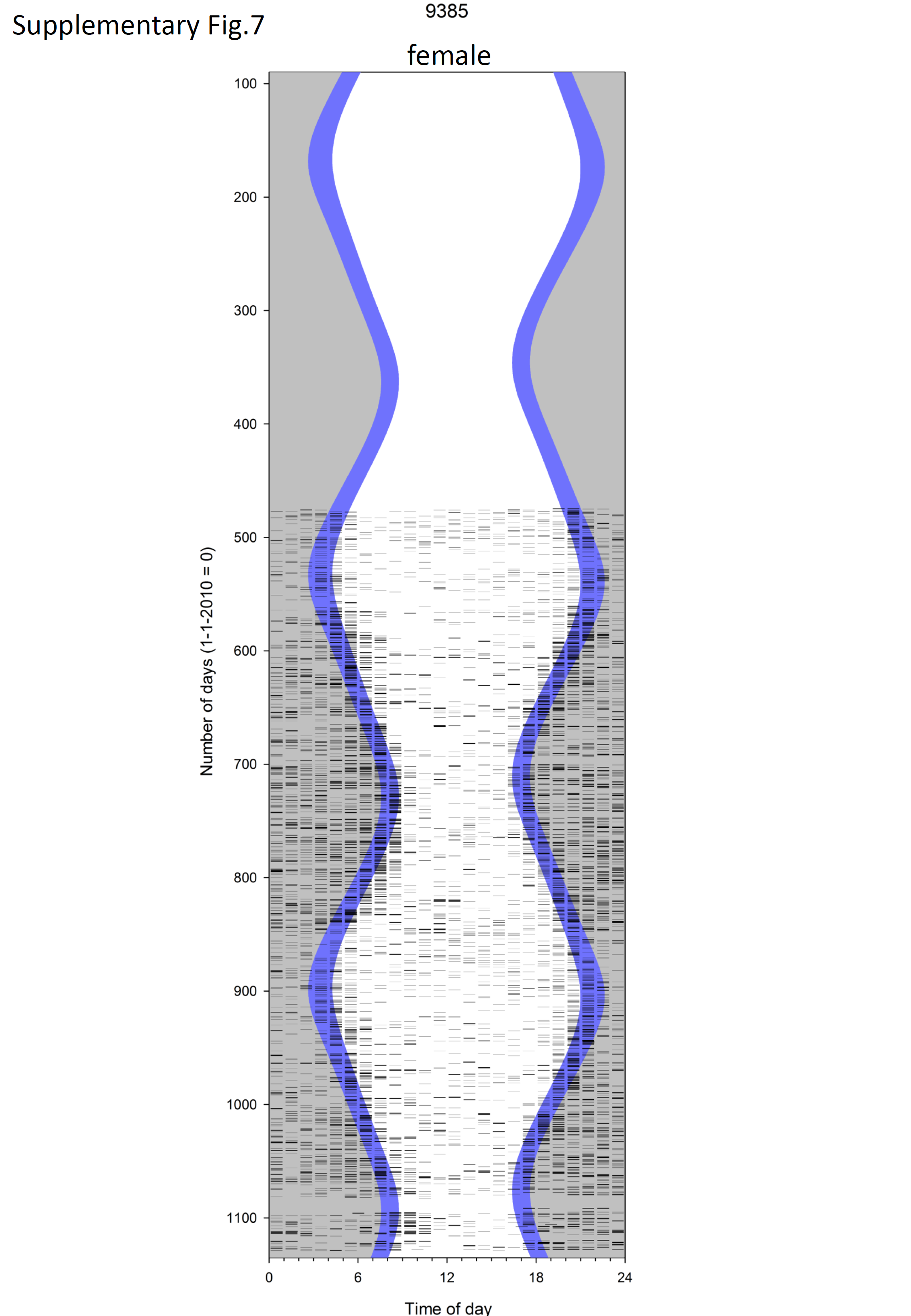

Supplement: Figure S7 — Actogram of Veluwezoom female 9385. See Fig.3 for explanation. (TIF) [file pone.0106997.s007.tif]

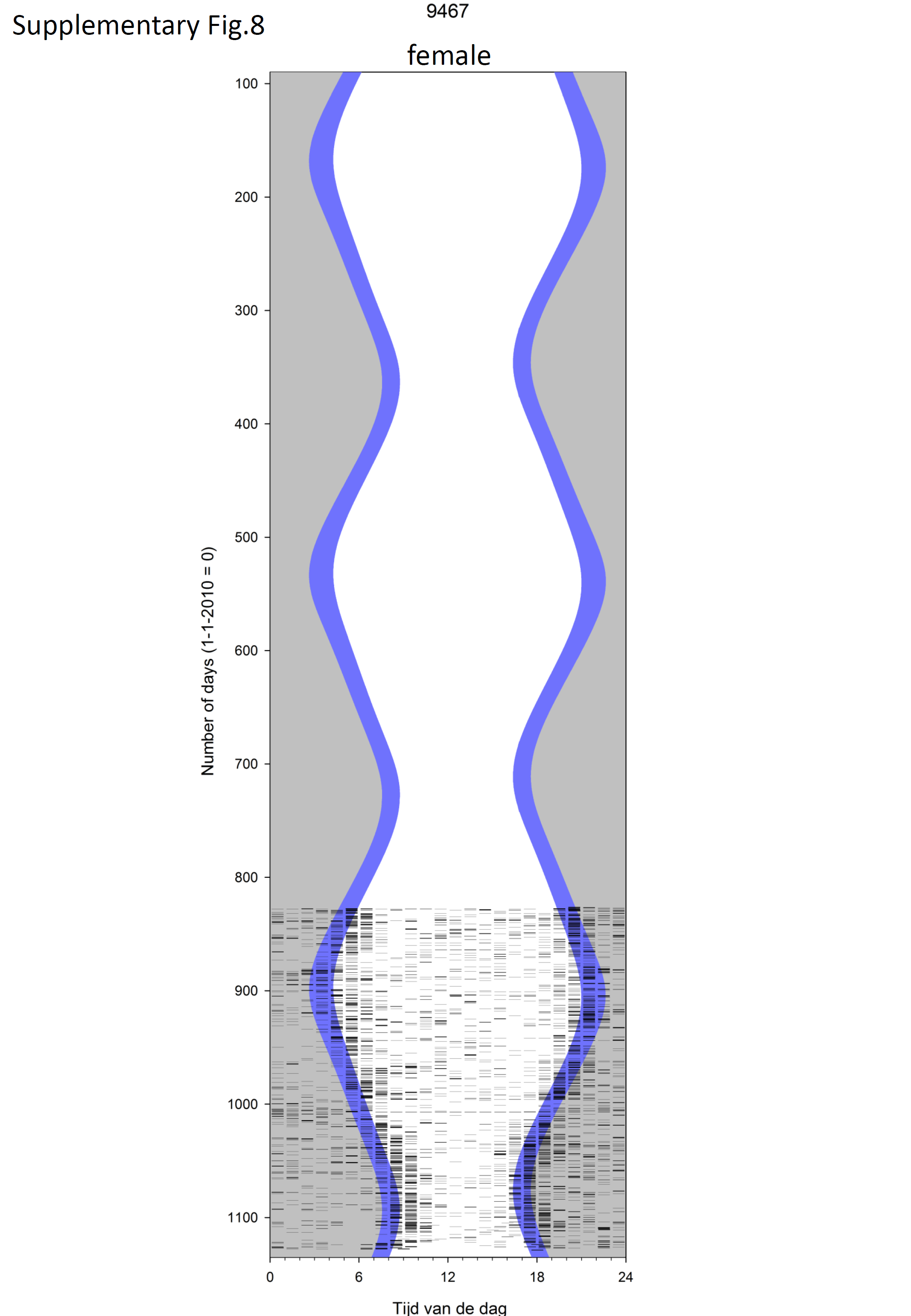

Supplement: Figure S8 — Actogram of Veluwezoom female 9467. See Fig.3 for explanation. (TIF) [file pone.0106997.s008.tif]

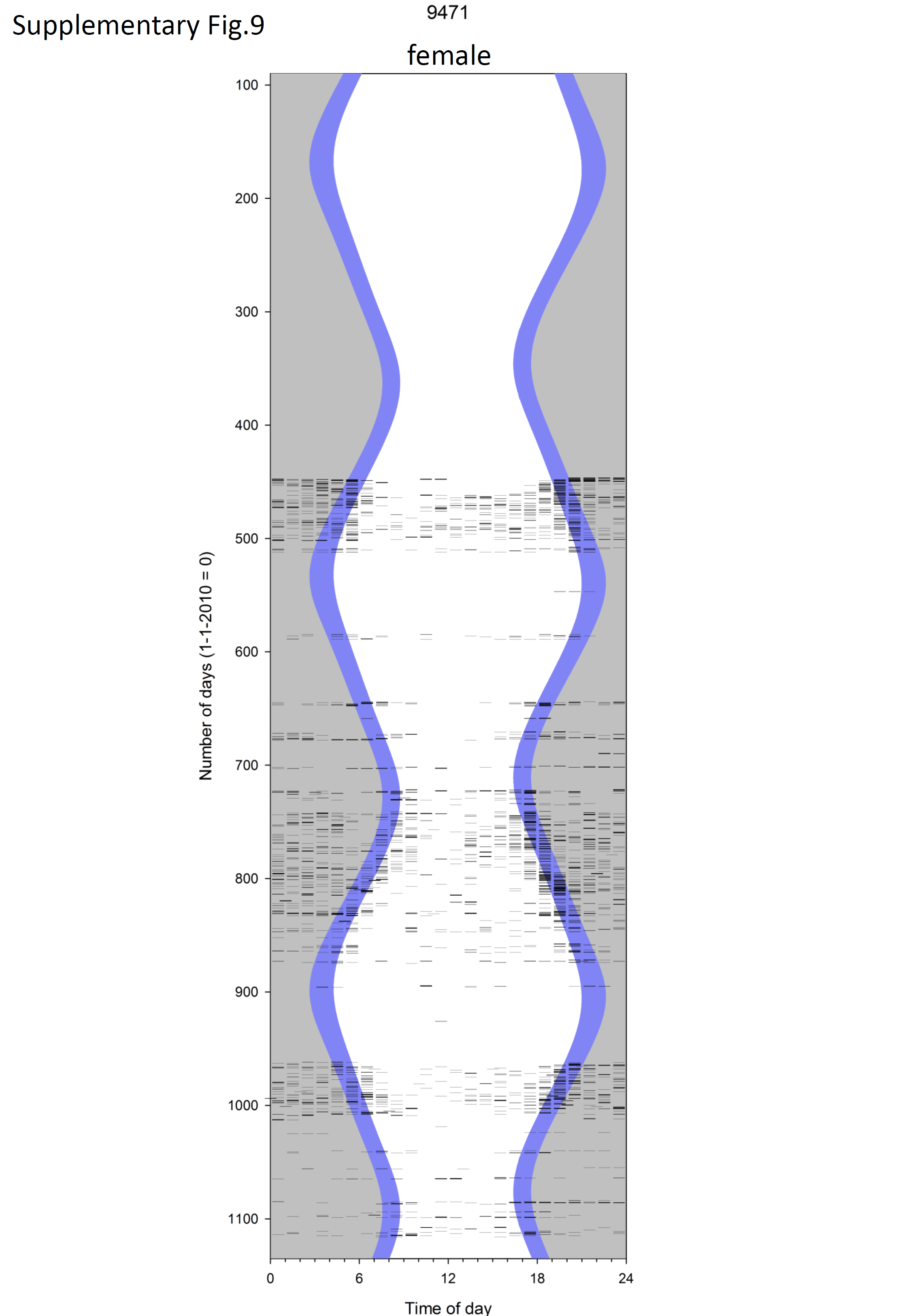

Supplement: Figure S9 — Actogram of Veluwezoom female 9471. See Fig.3 for explanation. (TIF) [file pone.0106997.s009.tif]
